# Improving Phenotypic Prediction by Combining Genetic and Epigenetic Associations

**DOI:** 10.1016/j.ajhg.2015.05.014

**Published:** 2015-07-02

**Authors:** Sonia Shah, Marc J. Bonder, Riccardo E. Marioni, Zhihong Zhu, Allan F. McRae, Alexandra Zhernakova, Sarah E. Harris, Dave Liewald, Anjali K. Henders, Michael M. Mendelson, Chunyu Liu, Roby Joehanes, Liming Liang, BIOS Consortium, Bastiaan T. Heijmans, Peter A.C. ’t Hoen, Joyce van Meurs, Aaron Isaacs, Rick Jansen, Lude Franke, Dorret I. Boomsma, René Pool, Jenny van Dongen, Jouke J. Hottenga, Marleen M.J. van Greevenbroek, Coen D.A. Stehouwer, Carla J.H. van der Kallen, Casper G. Schalkwijk, Cisca Wijmenga, Sasha Zhernakova, Ettje F. Tigchelaar, P. Eline Slagboom, Marian Beekman, Joris Deelen, Diana van Heemst, Jan H. Veldink, Leonard H. van den Berg, Cornelia M. van Duijn, Bert A. Hofman, André G. Uitterlinden, P. Mila Jhamai, Michael Verbiest, H. Eka D. Suchiman, Marijn Verkerk, Ruud van der Breggen, Jeroen van Rooij, Nico Lakenberg, Hailiang Mei, Maarten van Iterson, Michiel van Galen, Jan Bot, Peter van ’t Hof, Patrick Deelen, Irene Nooren, Matthijs Moed, Martijn Vermaat, Dasha V. Zhernakova, René Luijk, Marc Jan Bonder, Freerk van Dijk, Wibowo Arindrarto, Szymon M. Kielbasa, Morris A. Swertz, Erik W. van Zwet, Daniel Levy, Nicholas G. Martin, John M. Starr, Cisca Wijmenga, Naomi R. Wray, Jian Yang, Grant W. Montgomery, Lude Franke, Ian J. Deary, Peter M. Visscher

**Affiliations:** 1 Queensland Brain Institute, University of Queensland, Brisbane 4072, Australia; 2 University of Queensland Diamantina Institute, Translational Research Institute, University of Queensland, Brisbane, QLD 4072, Australia; 3 Department of Genetics, University Medical Center Groningen, University of Groningen, Groningen 9713 AV, the Netherlands; 4 Centre for Cognitive Ageing and Cognitive Epidemiology, University of Edinburgh, Edinburgh EH8 9JZ, UK; 5 Medical Genetics Section, Centre for Genomic and Experimental Medicine, Institute of Genetics and Molecular Medicine, University of Edinburgh, Edinburgh EH4 2XU, UK; 6 Queensland Institute of Medical Research Berghofer Medical Research Institute, Brisbane, QLD 4029, Australia; 7 Framingham Heart Study and Boston University School of Medicine, Boston, MA 01702, USA; 8 Department of Cardiology, Boston Children’s Hospital, Boston, MA 02115, USA; 9 Population Studies Branch, National Heart, Lung, and Blood Institute, NIH, Bethesda, MD 20892-7936, USA; 10 Department of Biostatistics, Boston University, Boston, MA 02118, USA; 11 Hebrew Senior Life, Harvard Medical School, Boston, MA 02131, USA; 12 Departments of Epidemiology and Biostatistics, T.H. Chan School of Public Health, Harvard University, Boston, MA 02115, USA; 13 Department of Psychology, University of Edinburgh, Edinburgh EH8 9JZ, UK

## Abstract

We tested whether DNA-methylation profiles account for inter-individual variation in body mass index (BMI) and height and whether they predict these phenotypes over and above genetic factors. Genetic predictors were derived from published summary results from the largest genome-wide association studies on BMI (n ∼ 350,000) and height (n ∼ 250,000) to date. We derived methylation predictors by estimating probe-trait effects in discovery samples and tested them in external samples. Methylation profiles associated with BMI in older individuals from the Lothian Birth Cohorts (LBCs, n = 1,366) explained 4.9% of the variation in BMI in Dutch adults from the LifeLines DEEP study (n = 750) but did not account for any BMI variation in adolescents from the Brisbane Systems Genetic Study (BSGS, n = 403). Methylation profiles based on the Dutch sample explained 4.9% and 3.6% of the variation in BMI in the LBCs and BSGS, respectively. Methylation profiles predicted BMI independently of genetic profiles in an additive manner: 7%, 8%, and 14% of variance of BMI in the LBCs were explained by the methylation predictor, the genetic predictor, and a model containing both, respectively. The corresponding percentages for LifeLines DEEP were 5%, 9%, and 13%, respectively, suggesting that the methylation profiles represent environmental effects. The differential effects of the BMI methylation profiles by age support previous observations of age modulation of genetic contributions. In contrast, methylation profiles accounted for almost no variation in height, consistent with a mainly genetic contribution to inter-individual variation. The BMI results suggest that combining genetic and epigenetic information might have greater utility for complex-trait prediction.

## Introduction

Obesity is a major risk factor for a number of chronic diseases, including diabetes, cardiovascular diseases, and cancer.^1–4^ Once considered a health burden only in high-income countries, it is a growing epidemic that is dramatically on the rise in low- and middle-income countries, particularly in urban settings. Knowledge of the genetic and environmental contributors to obesity is necessary for developing effective strategies to reduce its global burden. Body mass index (BMI) is a commonly used measure for quantifying obesity. Although many genetic determinants of BMI have been identified by large genome-wide association studies (GWASs),^5^ only about 10% of the inter-individual variation in BMI has been explained by genetic factors. With recent advances in high-throughput genomic technologies, researchers are now turning to epigenetics as a way of understanding the interplay between genetics and environment and their contribution to complex traits and diseases.

Epigenetics refers to the regulatory processes that control gene expression without altering the DNA sequence. The most studied epigenetic process is DNA methylation, the reversible addition of a methyl group primarily to a cytosine residue at a CpG dinucleotide. Because epigenetic variation reflects both genetic and environmental exposures, there is potential to identify novel disease-associated genes and pathways that might not be discovered through genetic studies alone. Methylome-wide association studies (MWASs), using methylation arrays such as the Illumina Infinium HumanMethylation450 array, have already begun to identify genomic CpG sites whose methylation levels are associated with BMI.^6^ DNA-methylation levels at specific CpG sites have already shown to be accurate predictors of age and smoking status,^7–9^ and such phenotypic prediction could extend to complex traits and disease and potentially improve prediction over genetic information. The ability of DNA-methylation profiles to predict cross-sectionally complex traits independently of genotypic information has not yet been explored. Here, we investigate whether DNA-methylation profiles associate with BMI and height independently of genotypic information. BMI and height represent two complex traits with different relative contributions of genetics and environment to inter-individual variance.^10–13^ Heritability estimates for BMI are high but vary (0.3–0.8) among twin and family studies. The genetic contribution appears to vary with age, such that it has a greater influence during childhood than during adult life.^10^ In contrast, height is known to have a mostly genetic contribution; heritability estimates from both twin and family studies are consistently around 0.8 in nutritionally replete societies.^11–13^ These findings suggest that epigenetic contributions might be greater for BMI than for height.

Therefore, the present study aimed to test the relative contributions of DNA-methylation status and genetic variation to inter-individual variation in BMI and height. We hypothesized a priori that after genetic determinants of phenotype are accounted for, DNA methylation will provide a far more substantial contribution to inter-individual variation in BMI than to variation in height. To this end, we first performed an MWAS for BMI and height in two independent datasets; the discovery sample comprised 1,366 older individuals from two Scottish birth cohorts (the Lothian Birth Cohorts [LBCs] of 1921 [n = 446; mean age 79.1 ± 0.6 years] and 1936 [n = 920; mean age 69.5 ± 0.8 years]), and the validation sample was the LifeLines DEEP cohort of Dutch adult individuals (n = 750; mean age 45.5 ± 13.3 years). For each trait, we generated methylation-profile scores (a weighted sum of the methylation levels at associated CpG sites) in the validation cohort on the basis of the observed CpG associations in the discovery cohort, and we estimated the proportion of height and BMI variance accounted for by these DNA-methylation profiles. We also determined whether the methylation-profile scores were associated with the two traits independently of genetic-profile scores (weighted sum of associated effect alleles of associated SNPs) on the basis of results from the most recent BMI and height meta-GWASs carried out by the Genetic Investigation of Anthropometric Traits (GIANT) consortium.^14^

In adults, the BMI cutoffs that define obesity are not linked to age and do not differ for men and women, whereas in children BMI varies with age and sex.^15^ Therefore, methylation changes associated with BMI in adults might not necessarily reflect those observed in children or adolescents. To investigate this further, we tested whether BMI-associated methylation changes observed in the adults of the LBCs and LifeLines DEEP cohort were predictive of BMI in adolescents from the Brisbane Systems Genetics Study (BSGS; n = 403; mean age 14.0 ± 2.4).

## Material and Methods

### Cohorts

#### LBCs

The LBCs comprise individuals born in 1921 (LBC1921) and 1936 (LBC1936), and most of these individuals were participants in the Scottish Mental Surveys (SMSs) of 1932 and 1947, respectively, when nearly all 11-year-old children in Scotland completed an IQ-type test in school. The LBC studies provide follow up of surviving SMS participants who are living in the Lothian region (Edinburgh city and outskirts) of Scotland.^16–18^ The LBC studies focus on the determinants of people’s cognitive aging differences and collect detailed information on cognitive, biomedical, lifestyle, socio-demographic, behavioral, physical, and psychological factors. An overview of the data collected in the LBCs can be found in the cohorts' profile article.^18^ The current study draws upon the baseline examinations (including blood-sample collection and phenotypic measurements) of 550 LBC1921 participants recruited in 1999–2001 (average age of 79 years) and 1,091 LBC1936 participants recruited in 2004–2007 (average age of 70 years).

#### LifeLines DEEP

This is a sub-cohort (n = 752, recruited in 2013) of the LifeLines study,^19^ the latter of which is a multi-disciplinary prospective population-based cohort study examining the health and health-related behaviors of 167,729 persons living in the north of the Netherlands in a unique three-generation design. It employs a broad range of investigative procedures in assessing the biomedical, socio-demographic, behavioral, physical, and psychological factors contributing to the health and disease of the general population and has a special focus on multi-morbidity and complex genetics. A full description of the LifeLines DEEP study can be found in the paper describing the cohort and data.^19^

#### BSGS

The BSGS is a study on adolescent twins comprising a total of 962 individuals from 314 families of European descent,^20^ and a subset of these individuals have DNA-methylation data (614 individuals from 177 families). Families consist of adolescent monozygotic (MZ) and dizygotic (DZ) twins, their siblings, and their parents. The BSGS comprises a sub-sample from a larger and continuing study on families with adolescent twins. Recruitment commenced in 1992. A full description of the BSGS cohort has been previously provided.^20,21^

### Ethics

Ethics permission for LBC1921 was obtained from the Lothian Research Ethics Committee (wave 1: LREC/1998/4/183). Ethics permission for LBC1936 was obtained from the Multi-Centre Research Ethics Committee for Scotland (wave 1: MREC/01/0/56) and the Lothian Research Ethics Committee (wave 1: LREC/2003/2/29). The BSGS was approved by the Queensland Institute for Medical Research Human Research Ethics Committee. The LifeLines DEEP study was approved by the ethical committee of the University Medical Centre Groningen (document no. METC UMCG LLDEEP: M12.113965). For all studies, written consent was obtained from all participants.

### Phenotypic Measurements

#### LBCs

Weight and height were measured in the LBCs by a trained nurse according to a standardized protocol. Participants were asked to remove their shoes before a seca stadiometer was used to assess height in centimeters. Weight (after participants removed shoes and outer clothing) was measured in kilograms by electronic seca scales, which provided digital readouts.

#### LifeLines DEEP

Height was measured without shoes by the seca 222 stadiometer. Weight was measured without shoes and heavy clothing by the seca 761 scale. All measurements were performed by a trained research nurse.

#### BSGS

Height and weight were both measured clinically with a stadiometer and accurate scales, respectively. Anthropometric measurements were only available for the offspring.

Complete blood cell counts (lymphocytes, monocytes, neutrophils, eosinophils, and basophils) were measured in the LBCs and LifeLines cohort.

### DNA Methylation

Whole-blood samples were collected at the same time as phenotypic measurements in all studies. Extracted DNA was profiled with the Infinium HumanMethylation450 BeadChip,^22^ and data were available on 752 LifeLines DEEP participants, 1,518 LBC participants (514 from LBC1921 and 1,004 from LBC1936), and 614 BSGS participants. For each of the LBCs and the LifeLines DEEP cohort, samples were randomized on 96-well plates, and methylation arrays were run in a single experiment to minimize batch effects. Low-quality probes and samples were excluded from further analysis as described below.

#### LBC DNA-Methylation Quality Control

Details of DNA extraction and methylation profiling are described elsewhere.^23^ Background correction of the raw intensity data and generation of the methylation beta values were done with the R minfi package. Quality-control (QC) steps included the removal of probes with a low (<95%) detection rate at p < 0.01. Array control probes were inspected manually, and low-quality samples (e.g., samples with inadequate hybridization, bisulfite conversion, nucleotide extension, or staining signal) were removed. Samples with a low call rate according to the Illumina-based threshold (samples with <450,000 probes detected at p < 0.01) were removed. LBC samples had been genotyped with the Illumina 610-Quadv1 genotyping platform. Genotype information from the 65 SNP control CpG probes on the methylation chip were cross-validated with those from the genotyping chip with the R wateRmelon package. Where there was low correspondence, samples were excluded (n = 9). We also excluded eight participants whose reported sex did not match their predicted sex according to methylation levels for probes on the X and Y chromosomes.

#### LifeLines DEEP DNA-Methylation QC

Details of DNA extraction and methylation profiling are described elsewhere.^19^ Probe QC, background correction, color correction, and normalization were performed with a custom pipeline based on the pipeline by Tost and Touleimat.^24^ All methylation probes were re-mapped to the human genome (hg37, UCSC Genome Browser),^25^ and both poorly mapping probes and probes with a SNP in the single-base extension side (according to GoNL^26^) were removed in the same step. Data were normalized with DASEN.^27^

#### BSGS DNA-Methylation QC

Details of DNA extraction, methylation profiling, and methylation QC are provided elsewhere.^21^

In all cohorts, non-autosomal probes and probes with underlying SNPs at the target CpG site (according to Illumina annotation) were excluded from further analysis. Methylation levels are presented as beta values, which range between 0 and 1, where a value of 0 indicates that all copies of the CpG site in the sample were completely unmethylated (no methylated molecules were measured), and a value of 1 indicates that every copy of the site was methylated. Beta values were then processed as follows in all cohorts. The beta values were logit transformed: log (beta/(1 − beta). For removal of variation due to batch effects and covariates, the logit-transformed beta values were regressed onto the technical variables (plate, array, and array position) and covariates (sex and age for the main analysis; in addition, cell count was adjusted in a sensitivity analysis in the LBCs and LifeLines DEEP cohort). Residuals from this linear regression were inverse-normal transformed and used in all subsequent analyses.

### Genotyping

Genotype data were available for all samples with DNA-methylation data in the three cohorts. The LBC and BSGS samples were genotyped with the Illumina Human610-Quad v1.0 genotyping platform, and data were available on all participants with DNA-methylation data. After QC, genotyped data were imputed with 1000 Genomes Phase 1 version 3^28^ and IMPUTE2.^29,30^ The LifeLines DEEP samples were genotyped with the HumanCytoSNP-12 BeadChip and the ImmunoChip,^31^ a customized Illumina Infinium array. The data were merged and subsequently imputed with GoNL^26,32^ and IMPUTE2.^29,30^ Details of QC in each cohort are described below.

#### LBC Genotyping QC

DNA samples from each individual were genotyped with the Illumina Human610-Quad BeadChip. Individuals were excluded on the basis of unresolved gender discrepancy, relatedness, call rate (≤0.95), and evidence of non-European descent. SNPs were included in the analyses if they met the following conditions: call rate ≥ 0.98, minor allele frequency ≥ 0.01, and Hardy-Weinberg equilibrium test with p ≥ 0.001.

#### LifeLines DEEP Genotyping QC

Details of DNA extraction, genotyping, and QC are provided elsewhere.^19^

#### BSGS Genotyping QC

DNA samples from each individual were genotyped by the Scientific Services Division at deCODE Genetics (Iceland) with the Illumina Human610-Quad BeadChip. Genotypes were called with the Illumina BeadStudio software. A detailed description of genotyping QC can be found elsewhere.^20,33^

### Methylome-wide Association Analysis in the LBCs and LifeLines DEEP Cohort

The BMI and height phenotypes were adjusted for sex and age and standardized for the generation of *Z* scores. Linear regression analysis was used to test the association between each CpG probe (independent variable) and the BMI or height *Z* score phenotype (dependent variable).

### Methylation-Profile Scores for BMI and Height

In the LBCs and LifeLines DEEP cohort, we first selected CpG probes on the basis of a Bonferroni-corrected association p value threshold (p < 0.05/[number of probes]). To remove redundant CpG probes from the methylation-profile score, if multiple probes passed the p value threshold and had a pairwise correlation greater than 0.1 within a 500-bp window, we selected only the most significant probe for the score. The choice of correlation threshold and window size was based on previous studies that investigated pairwise probe correlation as a function of the distance between probes.^34,35^ BMI and height methylation-profile scores were calculated as the weighted sum of the selected CpG methylation levels (the weights for each CpG probe were the effect sizes from the MWAS). We used selected probes and effect sizes from the LBC MWAS to generate a methylation-profile score in the LifeLines DEEP cohort, and vice versa.

For BMI, as a secondary replication cohort, we generated an additional methylation-profile score, whereby we selected probes on the basis of results from a larger, independent MWAS on BMI in the Framingham Heart Study (n = 2,377; mean age 67 ± 9 years; age range = 40–93 years; M.M.M., unpublished data). In this analysis, 78 CpG probes had an association p value < 1.22 × 10^−7^ (Bonferroni correction for 409,403 probes) and were selected for generating a BMI methylation-profile score. To generate a Framingham-based methylation score in the LBCs, we derived effect sizes for these 78 probes from the LifeLines DEEP MWAS, whereas we derived effect sizes from the LBC MWAS to generate the score in the LifeLines DEEP cohort.

### Genetic-Profile Score for BMI and Height

We used SNP genotype data to calculate genetic-profile scores for BMI and height. SNPs and weights (effect sizes) used for generating the genetic-profile scores (the weighted sum of the effect allele count) were based on the GIANT meta-GWAS for BMI in ∼350,000 individuals^14^ and for height in ∼250,000 individuals.^36^ It is important to note that none of the LBC, LifeLines DEEP, or BSGS participants were part of the GIANT meta-GWAS, so discovery bias was not an issue. Prior testing in an independent cohort indicated that using all HapMap3 SNPs provided the best predictor for BMI,^14^ whereas SNPs that had a p value < 5 × 10^−5^ and that were selected with the GCTA-COJO (conditional and joint genome-wide analysis) function in the GCTA software^37^ provided the best predictor for height.^14,36^

### Proportion of Phenotypic Variance Explained in the LBCs and LifeLines DEEP Cohort

Using linear regression, in each cohort we estimated how much variance in the sex- and age-adjusted BMI and height phenotypes (adjusted R^2^) was explained by the methylation- and genetic-profile scores, both individually and combined. We also looked for any evidence of interaction between the methylation- and genetic-profile scores. For each trait in each cohort, we ran the following four regression models and extracted the proportion of variance explained from each:

Model 1: trait ∼ MWAS score
Model 2: trait ∼ GWAS score
Model 3: trait ∼ MWAS score + GWAS score
Model 4: trait ∼ MWAS score + GWAS score + (MWAS score × GWAS score)

We used an ANOVA to test whether the interaction model (model 4) explained significantly more of the variation in the phenotype than the additive model (model 3). A summary of the cross-cohort GWAS and MWAS predictions is presented in Table 1.

### Proportion of Variance Explained in BMI of Adolescent Individuals

We generated five methylation scores in the BSGS individuals and report how much of the variation in sex- and age-adjusted BMI was explained by each of the scores: (1) probe selection and weights derived from the LBCs; (2) probe selection and weights derived from the LifeLines DEEP cohort (3); probe selection from the Framingham discovery and weights derived from the LBCs; (4) probe selection from the Framingham discovery and weights derived from the LifeLines DEEP cohort; and (5) probe selection and weights derived from a fixed-effect meta-analysis of the LBCs and LifeLines DEEP cohort. To estimate the proportion of variance accounted for by the profile scores in the BSGS cohort, we corrected the sex- and age-adjusted BMI *Z* scores (scores standardized within the cohort) for family structure by using a linear mixed model (LMM) analysis in GCTA,^38^ in which we used SNP genotypes to estimate pedigree relatedness (pairwise relatedness < 0.05 was set to 0 according to the method in Zaitlen et al.^39^). Residuals from this LMM analysis were used as the sex-, age-, and family-structure-corrected BMI phenotype. The proportion of variance explained in the latter phenotype by each of the abovementioned methylation-profile scores was estimated by linear regression.

### Correcting for Cell Count

Chronic inflammation is known to be associated with obesity, and white blood cell counts have been shown to increase with increasing BMI.^40^ Although the aim of this study was to investigate how much variation in BMI and height is captured by genetic and methylation differences irrespective of causality, we did perform the above analyses on cell-count-corrected methylation data as a sensitivity analysis.

## Results

### Cohort Characteristics

After sample QC, 1,366 samples from the LBCs (n = 446 from LBC1921 and n = 920 from LBC1936), 752 samples from the LifeLines DEEP cohort, and 403 samples from the BSGS cohort (after we removed one individual from each MZ twin pair) had methylation, phenotype, and genotype data. Cohort characteristics of these samples are provided in Table 2. The LifeLines DEEP participants had a much wider age range (18–81 years) and were on average much younger (mean 45.5 ± 13.3 years) than LBC participants (69.5 ± 0.8 years in LBC1936 and 79.1 ± 0.6 years in LBC1921). The mean age in the BSGS cohort was 14 ± 2.4 years. BMI and height distributions for each cohort are shown in Figures S1 and S2. The BMI and height phenotypes were adjusted for age and sex in each cohort.

### Methylome-wide Association Analysis

To create a multi-probe methylation predictor, we first conducted a methylome-wide association analysis. A total of 431,951 and 407,935 CpG probes remained in the LBC and LifeLines DEEP datasets, respectively, after QC and probe filtering. Probes with an association p value < 1.16 × 10^−7^ in the LBC dataset and a p value < 1.22 × 10^−7^ in the LifeLines DEEP dataset were considered to be significantly associated after Bonferroni correction for the number of probes tested. After removal of correlated probes, nine CpG probes in the LBC dataset and five probes in the LifeLines DEEP dataset were associated with BMI and were used for generating methylation-profile scores (Table S1). Two probes (cg06500161 and cg11024682) were significantly associated with BMI in both cohorts—cg06500161 is found in an intronic region of *ABCG1* (ATP-binding cassette, sub-family G, member 1 [MIM: 603076]), and cg11024682 is intronic to one isoform of *SREBF1* (sterol regulatory element binding transcription factor 1 [MIM: 184756]). Both genes are known to be involved in lipid metabolism, but neither has been identified by GWASs to harbor genetic variants that are associated with BMI.

For height, no CpG probes passed the p value threshold in the LBCs, whereas only a single probe passed the threshold in the LifeLines DEEP cohort. Therefore, to generate a height-profile score, we used a less stringent association p value of <0.001 for probe selection. 507 and 949 CpG probes were selected in the LBCs and LifeLines DEEP cohort, respectively. Quantile-quantile plots for each MWAS are shown in Figure S3. We observed inflation in the lambda values—for BMI, lambdas were 1.53 and 1.17 in the LBCs and LifeLines DEEP cohort, respectively, whereas for height, lambdas were 1.12 and 1.36, respectively. Lambdas close to 1 (SD = 0.1) were observed with permutation analysis (performed in both the LBCs and LifeLines DEEP cohort), which indicates that the inflation was due to real signal and not an artifact of our assumption of the null distribution of the test statistic.

### Proportion of BMI and Height Variance Explained by Profile Scores in the LBCs and LifeLines DEEP Cohort

**Figure 1:**
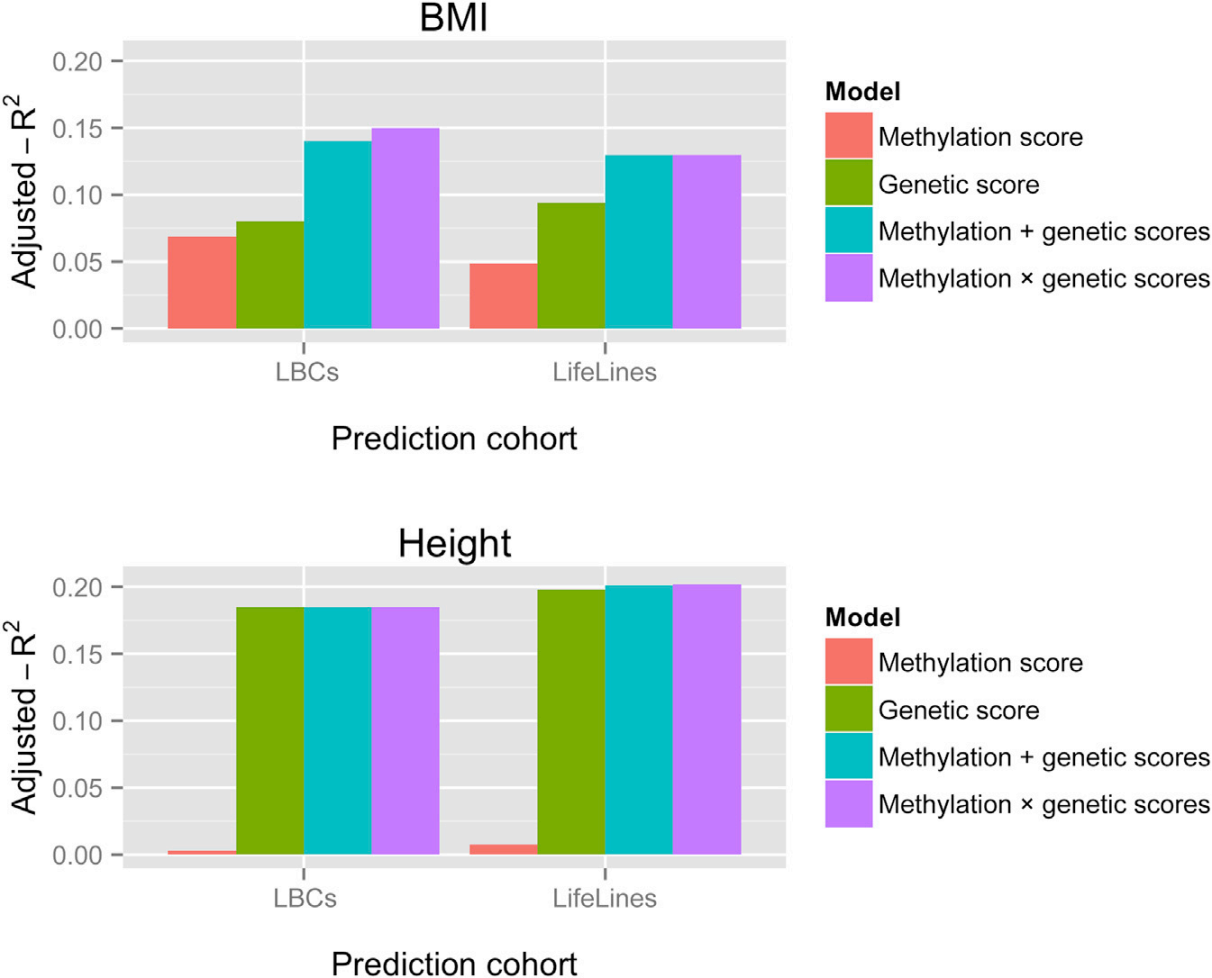
BMI and Height Prediction The plots depict how much of the variance in the sex- and age-adjusted BMI and height phenotypes (adjusted R^2^) was explained by the methylation-profile score, the genetic-profile score, an additive model including both scores (methylation + genetic), and an interaction model (methylation × genetic). The methylation score in the LBCs is based on selected probes and effects sizes from the LifeLines DEEP MWAS, and vice versa. The genetic-profile scores are based on results from the GIANT meta-GWAS.

Consistent with expectation, all methylation- and genetic-profile scores were correlated with their respective traits in the anticipated direction (Table S2). The methylation-profile scores explained 6.9% and 4.9% (p value < 1 × 10^−15^ and 7 × 10^−10^, respectively) of the variation in BMI in the LBCs and LifeLines DEEP cohort, respectively, whereas the genetic-profile scores explained 8.0% and 9.4% (p value < 1 × 10^−15^), respectively (Figure 1). When both the methylation- and genetic-profile scores were included in an additive model for BMI, each remained independently associated with BMI. The proportion of variance explained by the additive model was 14.0% and 13.6% in the LBCs and LifeLines DEEP cohort, respectively, suggesting a mainly additive effect of the two scores on BMI (Figure 1).

The BMI methylation-profile scores, based on 78 probes selected from an MWAS in the larger Framingham Heart Study (M.M.M., unpublished data) but weighted with effect sizes estimated in the LBCs, explained 7.3% of the variation in BMI in the LifeLines DEEP cohort, whereas a profile score based on the effects estimated in the LifeLines DEEP cohort explained 11% of the variation in the LBCs. As before, the methylation-profile scores showed an additive effect with the genetic-profile scores (Figure S4). Compared to the methylation-profile scores derived from the MWAS in the LBCs or LifeLines DEEP cohort, the larger R^2^ values for the profile scores based on probes identified in the Framingham cohort suggest that the larger sample size in the latter study provided more power to identify additional CpG probes and hence allowed us to explain a higher proportion of variance in BMI.

The height methylation-profile scores were associated with height and explained 0.31% and 0.76% (p value = 0.02 and 0.01 of the variation in the LBCs and LifeLines DEEP cohort, respectively). The height genetic-profile scores explained 18.5% and 19.8% (p value < 1 × 10^−15^) of the inter-individual variation in the height phenotype in the LBCs and LifeLines DEEP cohort, respectively (Figure 1). The additive model including both methylation- and genetic-profile scores explained 18.5% and 20.1% of the variation in the height phenotype in the LBCs and LifeLines DEEP cohort, respectively. However, the methylation-profile score showed no independent association in the LBCs (p = 0.16) and remained only marginally associated (p = 0.035) with the height phenotype independently of the genetic-profile score in the LifeLines DEEP cohort.

For BMI, the interaction model explained a slightly larger proportion of variance than did the additive model in the LBCs (15% versus 14%; ANOVA p value = 5 × 10^−6^) but not in the LifeLines DEEP cohort (Table S3). There was no significant interaction between the genetic- and methylation-profile scores for height in either cohort.

### Proportion of BMI Variance Explained in BSGS Adolescent Individuals

**Figure 2:**
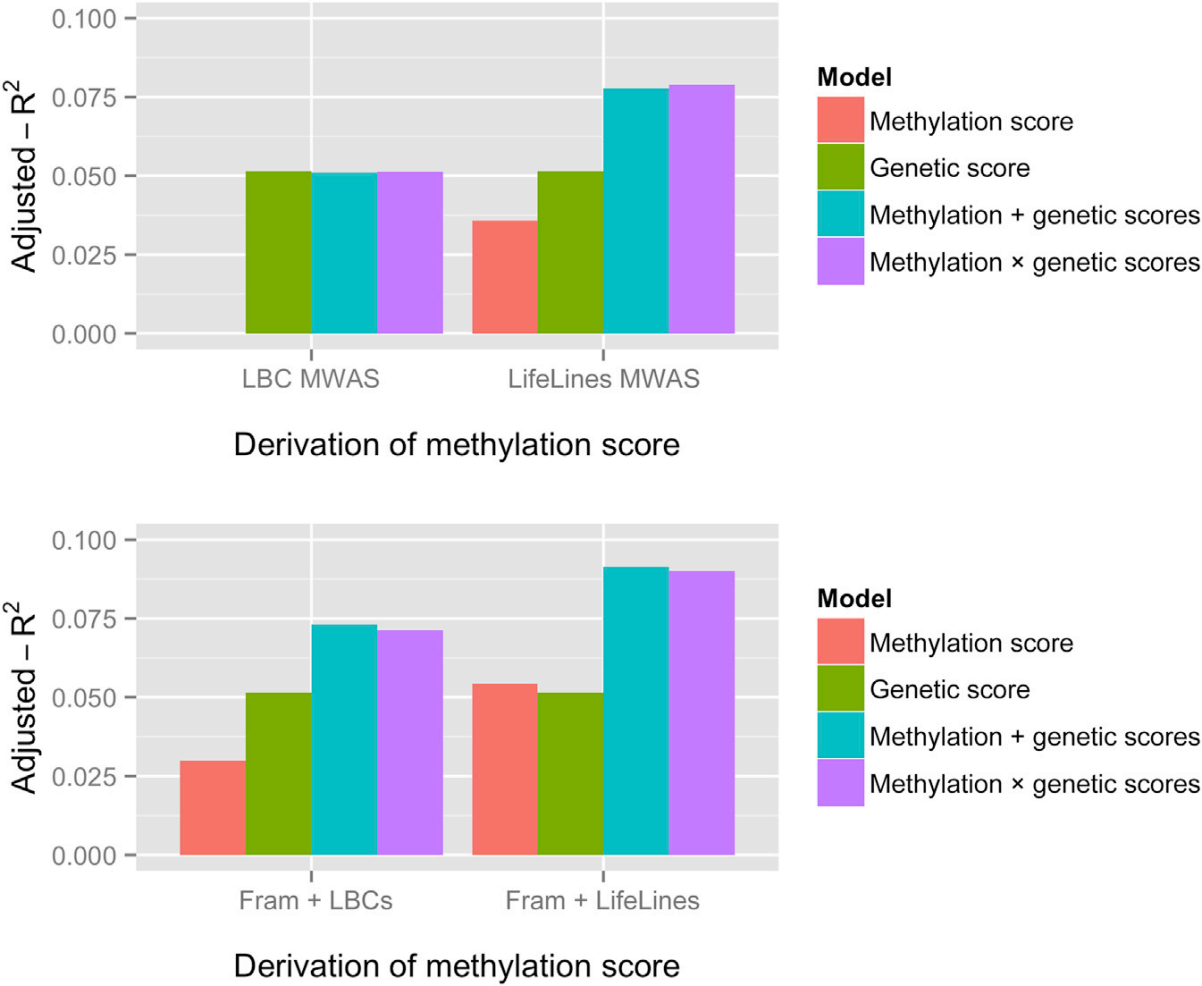
BMI Prediction in BSGS Adolescents The plots show how much of the variance in the sex- and age-adjusted BMI phenotype (adjusted R^2^) was explained by the methylation-profile score, the genetic-profile score, an additive model including both scores (methylation + genetic), and an interaction model (methylation × genetic). The GWAS scores are based on results from the GIANT meta-GWAS. Methylation scores are based on probe selection and weights derived from the LBCs MWAS or the LifeLines DEEP MWAS (upper panel) or probe selection from the Framingham discovery with weights derived from the LBCs or LifeLines DEEP studies (lower panel).

The methylation-profile scores derived from the MWAS analysis in the LBC individuals did not explain any variation (adjusted R^2^ = −0.001) in the sex- and age-adjusted BMI phenotype from the BSGS cohort, whereas that derived from the mostly middle-aged individuals of the LifeLines DEEP study explained 3.6% (p value = 8 × 10^−5^; Figure 2). Methylation scores based on the CpG probes identified in the larger Framingham MWAS but weighted with effect sizes from the older LBC individuals explained 3.0% of the variation in BMI in adolescent individuals. Based on the same CpG probes but effect sizes derived from the younger, albeit smaller, LifeLines DEEP cohort, the methylation-profile scores explained almost twice (5.4%) the variation in BMI in adolescent individuals (Figure 2).

Given that the proportion of variance explained in a prediction setting is a function of sample sizes of the discovery cohorts, the R^2^ values from different-sized cohorts are not directly comparable. We therefore compared the ratio of the methylation score R^2^ to the genetic score R^2^ to look at the relative contribution of the methylation- and genetic-profile scores to variance in BMI in both BSGS adolescents and older cohorts. As shown in Table S4, in all cases, the methylation-profile scores had a lower contribution to BMI variance in the BSGS cohort than in the other cohorts. The methylation predictor derived from older individuals (probes and weights for the methylation-profile score derived from the LBC MWAS) performed the worst.

A BMI methylation score based on a fixed-effect meta-analysis of the LBC and LifeLines DEEP MWAS results, whereby a Bonferroni correction for 374,629 common probes in the two cohorts (p value < 1.33 × 10^−7^) was used for selecting probes, performed better than the methylation score based on the LBC MWAS. However, despite the larger sample size, it performed worse than the predictor based on the LifeLines DEEP MWAS: its adjusted R^2^ was 0.028 (p value = 4.0 × 10^−4^).

### Correcting for Cell Count

In the LBCs, all cell counts except neutrophils were associated with sex- and age-adjusted BMI (p < 0.05), but only monocytes were associated with sex- and age-adjusted height. In contrast, in the LifeLines DEEP cohort, all cell counts were significantly associated with BMI, but not with height. Adjusting for cell count reduced some of the inflation observed in the uncorrected analysis—for BMI, lambdas were 1.28 and 1.00 in the LBCs and LifeLines DEEP cohort, respectively, whereas for height, lambdas were 1.00 and 1.15, respectively. The proportion of variance explained by the methylation scores after cell-count correction is shown in Figure S5. The cell-count-corrected methylation scores based on the MWAS discovery in the LBCs and LifeLines DEEP cohort remained significantly associated with BMI and showed an additive effect, although the proportion of variance explained was substantially less in the LBCs (3.2%). For height, the methylation-profile score was still marginally associated with the sex- and age-adjusted height phenotype in the LifeLines DEEP cohort (adjusted R^2^ = 0.0041; p value = 0.045), but not in the LBCs.

## Discussion

We investigated two traits that we postulated a priori to have varying contributions of genetic and environmental factors to inter-individual variability—we hypothesized that height would have a mostly genetic component, whereas BMI would have a larger environmental contribution that increases with age.^10^ We found that the methylation-profile scores contributed almost nothing to the inter-individual variance in height but showed a strong association with BMI. The BMI methylation-profile score improved prediction of BMI over and above the genetic-profile score. The two profile scores acted mostly in an additive manner, suggesting that methylation-profile scores capture information that is largely independent of the genetic determinants of BMI. Our results suggest that even if there are genetic variants whose effects on BMI are mediated by methylation, their contribution is small. Therefore, methylation profiles might have important utility in improving phenotype prediction over and above genetic data alone.

Furthermore, BMI methylation profiles in older people (the LBC individuals) did not predict well in adolescents (BSGS cohort). A methylation predictor based on CpG probes identified in a larger, independent study (Framingham) explained almost double the proportion of variance in BMI in BSGS adolescent individuals when the effect sizes used for generating the methylation-profile score were derived from the younger LifeLines DEEP cohort than when they were derived from the older LBC individuals. A methylation predictor based on the meta-analysis of the LBCs and LifeLines DEEP cohort, despite the larger sample size, performed worse than a predictor based on the LifeLines DEEP cohort alone. The relative contribution of the methylation and genetic predictors for BMI in adolescent individuals was also found to be much lower. Combined, the results suggest that these differences might be due to the direct effect of more prolonged exposure to environmental factors in older individuals, or the fact that older individuals are “exposed” to the phenotype for longer, and therefore might show larger effects on methylation due to reverse causation (Figure S6).

The effect sizes for individual CpGs are much larger than effect sizes for individual SNPs, and this is reflected in the fact that the proportion of variance explained by the CpGs identified in relatively small sample sizes (<1,500 individuals) is comparable to that explained by SNPs identified in very large samples used in genetic discovery (over 250,000 individuals). This suggests that bigger studies might be able to identify epigenetic variation that accounts for a larger proportion of the inter-individual variance of a complex trait.

A permutation analysis gives an indication of the highly correlated structure of the methylation probes in the genome. If lambda is the mean χ^2^ statistic across all ∼400,000 probes, then its sampling variance is 2/M, where M is the effective number of independent probes, i.e., the number of independent probes that give the same sampling variance as the observed variance. The SD of the genome-wide lambda from permutations therefore implies a surprisingly small effective number of independent methylation probes of only 2/0.1^2^ = 200, consistent with a complex correlation structure. Such a complex correlation structure or small effective number of probes does not imply the absence of meaningful and genome-wide biological inference, as shown, for example, for gene expression, which is also characterized by a complex correlation structure.^41^

A limitation of our study was the relatively small sample size of the LBCs and LifeLines DEEP cohort. We showed that a methylation-profile score based on a more extensive CpG probe list identified from the larger Framingham study performed the best. This suggests that the smaller sample size of the LBCs and LifeLines DEEP cohort lacked the power for statistical identification of additional CpGs. A sensitivity analysis using different p value thresholds to select CpG probes in the LBCs and LifeLines DEEP cohort showed that the ability of the methylation score to predict BMI decreased as the p value threshold was relaxed (Table S5). Forming large consortia to enable meta-analyses of multiple studies will overcome power issues and identify more robust associations, as well as estimate effect sizes more accurately. However, sample characteristics of the cohorts would need careful consideration for methylation analyses.

As more BMI-associated CpG sites are identified, the interaction between methylation and genetic profiles might become stronger, because it would be reasonable to expect that methylation at some of these CpGs might lie in the causal pathway, downstream of SNP effects. Further analysis to identify SNPs associated with both BMI and methylation levels at BMI-associated CpG sites would be needed to dissect the observed interaction and determine causality. Current work using a Mendelian randomization approach to identify a causal SNP (rs4925108) that is associated with methylation at a CpG site in *SREBF* suggests that both the SNP and the methylation levels at the CpG appear to be associated with BMI (M.M.M., unpublished data).

Another limitation of our study was the use of methylation profiles observed in blood. It is well known that tissue-specific DNA-methylation profiles exist; therefore, methylation profiles in blood might not be entirely representative of other tissues. If the primary interest for identifying epigenetic profiles is to determine causality, the tissue under investigation might be of great importance, and a more relevant tissue, such as adipose tissue, might be more suitable for a trait such as BMI or obesity. This might not apply for prediction, and comparing blood-derived methylation predictors with those derived from other tissues would be a logical next step if data were available.

The SNP arrays used in the BMI and height GWASs provide comprehensive coverage of the genome: 93% of common SNPs (both coding and non-coding) in the CEU population (Utah residents with ancestry from northern and western Europe from the CEPH collection) are tagged at r^2^ ≥ 0.8.^42^ In comparison, although the Infinium HumanMethylation450 array comprehensively evaluates promoter regions and CpG islands, as well as other potentially relevant intergenic regions, such as regulatory regions,^43^ it only interrogates a small subset of the ∼28 million CpG sites in the human genome. Therefore, other CpG sites might be missed, potentially giving an incomplete and biased view of the relative contribution of genetic and epigenetic factors to phenotypic variation. Despite this, the array has already proven to be a useful high-throughput technology for unraveling interesting biology: a number of studies have successfully identified CpGs in or near likely candidate genes associated with various phenotypes.

A drawback of using epigenetic disease markers, like any other molecular biomarker, is that they are vulnerable to confounding and reverse causation. This also applies to cell counts as a biomarker. The observed attenuation of the BMI variance explained by the methylation predictor when the MWAS was adjusted for cell counts suggests that both methylation and cell counts are involved in either the cause or the consequence of BMI differences between individuals. Distinguishing methylation changes that lie in the causal pathway from those that are a consequence of disease is an important task for understanding disease etiology and identifying new drug targets. Combining genetic and epigenetic data in a typical Mendelian randomization analysis might identify causal methylation changes due to genetic variation. In the context of BMI, methylation changes due to obesity would still be of interest for understanding the etiology of downstream disease outcomes, such as cardiovascular disease or type 2 diabetes. However, neither causality nor functional knowledge is necessary for prediction and was therefore not the focus of this study.

In summary, we have shown that inter-individual differences in environment or lifestyle are partly reflected in DNA-methylation data, and therefore DNA-methylation profiles have the potential to significantly improve complex-trait prediction over and above that of genetic predictors. Outside of disease association, applying accurate prediction of complex traits by using genetic and epigenetic predictors might be useful in forensic investigations where a biological sample is available but where there is no profile from the person whose sample is investigated.

## Consortia

The members of the BIOS Consortium are Bastiaan T. Heijmans, Peter A.C. 't Hoen, Joyce van Meurs, Aaron Isaacs, Rick Jansen, Lude Franke, Dorret I. Boomsma, René Pool, Jenny van Dongen, Jouke J. Hottenga, Marleen M.J. van Greevenbroek, Coen D.A. Stehouwer, Carla J.H. van der Kallen, Casper G. Schalkwijk, Cisca Wijmenga, Sasha Zhernakova, Ettje F. Tigchelaar, P. Eline Slagboom, Marian Beekman, Joris Deelen, Diana van Heemst, Jan H. Veldink, Leonard H. van den Berg, Cornelia M. van Duijn, Bert A. Hofman, André G. Uitterlinden, P. Mila Jhamai, Michael Verbiest, H. Eka D. Suchiman, Marijn Verkerk, Ruud van der Breggen, Jeroen van Rooij, Nico Lakenberg, Hailiang Mei, Maarten van Iterson, Michiel van Galen, Jan Bot, Peter van 't Hof, Patrick Deelen, Irene Nooren, Matthijs Moed, Martijn Vermaat, Dasha V. Zhernakova, René Luijk, Marc Jan Bonder, Freerk van Dijk, Wibowo Arindrarto, Szymon M. Kielbasa, Morris A. Swertz, and Erik W. van Zwet.

## Figures and Tables

**Table 1:** Summary of MWAS and GWAS Prediction Analyses

**Trait**	**Cohort**			**Location of Results**
**Probe Selection**	**Effect-Size Estimation**	**Prediction**
**MWAS Prediction**				

BMI	LifeLines DEEP	LifeLines DEEP	LBC	Figure 1
BMI	LBC	LBC	LifeLines DEEP	Figure 1
BMI	Framingham	LifeLines DEEP	LBC	Figure S4
BMI	Framingham	LBC	LifeLines DEEP	Figure S4
BMI	LifeLines DEEP	LifeLines DEEP	BSGS	Figure 2
BMI	LBC	LBC	BSGS	Figure 2
BMI	Framingham	LifeLines DEEP	BSGS	Figure 2
BMI	Framingham	LBC	BSGS	Figure 2
Height	LifeLines DEEP	LifeLines DEEP	LBC	Figure 1
Height	LBC	LBC	LifeLines DEEP	Figure 1

**GWAS Prediction**				

BMI	GIANT 2015	GIANT 2015	LBC	Figure 1
BMI	GIANT 2015	GIANT 2015	LifeLines DEEP	Figure 1
BMI	GIANT 2015	GIANT 2015	BSGS	Figure 2
Height	GIANT 2014	GIANT 2014	LBC	Figure 1
Height	GIANT 2014	GIANT 2014	LifeLines DEEP	Figure 1

**Table 2:** Cohort Characteristics of the LBC and LifeLines DEEP Participants at the Time of DNA-Methylation Assays

	**Cohort**			
**LBC1936**	**LBC1921**	**LifeLines DEEP**	**BSGS**
n	920	446	752	403
Age (years)	69.5 ± 0.8	79.1 ± 0.6	45.5 ± 13.3	14.0 ± 2.4
Female	49.5%	60.5%	57.8%	48.1%
BMI (kg/m^2^)	27.8 ± 4.4	26.2 ± 4.0	25.4 ± 4.2	20.4 ± 3.7
Height (cm)	166.4 ± 8.9	163.1 ± 9.3	175.2 ± 8.9	159.3 ± 11.6

## Accession Numbers

The European Genome-phenome Archive (EGA) accession number for the Lothian Birth Cohort methylation data reported in this paper is EGA: EGAS00001000910. The Database of Genotypes and Phenotypes (dbGaP) accession number for the Framingham Heart Study methylation data reported in this paper is dbGaP: phs000724.v2.p9. The NCBI Gene Expression Omnibus (GEO) accession number for the Brisbane Systems Genetic Study methylation data reported in this paper is GEO: GSE56105.

## Web Resources

The URLs for data presented herein are as follows:

Database of Genotypes and Phenotypes (dbGaP), http://www.ncbi.nlm.nih.gov/gap
European Genome-phenome Archive (EGA), https://www.ebi.ac.uk/ega/home
Gene Expression Omnibus (GEO), http://www.ncbi.nlm.nih.gov/geo/

## Supplemental Data

Document S1. Supplemental Acknowledgments, Figures S1–S6 and Tables S1–S5

Document S2. Article plus Supplemental Data
