# Supplementary material for: Improving Phenotypic Prediction by Combining Genetic and Epigenetic Associations
Source: Am J Hum Genet. 2015 Jul 2;97(1):75–85. doi: 10.1016/j.ajhg.2015.05.014 (PMC4572498; doi:10.1016/j.ajhg.2015.05.014)
Supplement: Document S1. Supplemental Acknowledgments, Figures S1–S6 and Tables S1–S5 [file mmc1.pdf]

The American Journal of Human Genetics
Supplemental Data

Improving Phenotypic Prediction by Combining Genetic and Epigenetic Associations

Sonia Shah, Marc J. Bonder, Riccardo E. Marioni, Zhihong Zhu, Allan F. McRae, Alexandra Zhernakova, Sarah E. Harris, Dave Liewald, Anjali K. Henders, Michael M. Mendelson, Chunyu Liu, Roby Joehanes, Liming Liang, BIOS Consortium, Daniel Levy, Nicholas G. Martin, John M. Starr, Cisca Wijmenga, Naomi R. Wray, Jian Yang, Grant W. Montgomery, Lude Franke, Ian J. Deary, and Peter M. Visscher

Supplemental Acknowledgments

We thank the cohort participants and team members who contributed to these studies.

Phenotype collection in the Lothian Birth Cohort 1921 was supported by the UK's Biotechnology and Biological Sciences Research Council (BBSRC), The Royal Society and The Chief Scientist Office of the Scottish Government. Phenotype collection in the Lothian Birth Cohort 1936 was supported by Age UK (The Disconnected Mind project). Methylation typing was supported by Centre for Cognitive Ageing and Cognitive Epidemiology (Pilot Fund award), Age UK, The Wellcome Trust Institutional Strategic Support Fund, The University of Edinburgh, and The University of Queensland. REM, SEH, DL, JMS, IJD and PMV are members of the University of Edinburgh Centre for Cognitive Ageing and Cognitive Epidemiology (CCACE). CCACE is supported by funding from the BBSRC, the Medical Research Council (MRC), and the University of Edinburgh as part of the cross-council Lifelong Health and Wellbeing initiative (MR/K026992/1). Research reported in this publication was supported by National Health and Medical Research Council (NHMRC) project grants 613608, APP496667, APP1010374 and APP1046880. NHMRC Fellowships to GWM, PMV, and NRW and Australia Research Council (ARC) Future Fellowship to NRW (FT0991360). The content is solely the responsibility of the authors and does not necessarily represent the official views of the NHMRC or ARC. LF was financially supported by grants from the Netherlands Organization for Scientific Research (NWO-VENI grant 916-10135 and NWO VIDI grant 917-14374) and a Horizon Breakthrough grant from the Netherlands Genomics Initiative (grant 92519031). The research leading to these

results has received funding from the European Community's Health Seventh Framework Programme (FP7/2007– 2013) under grant agreement no. 259867. The Framingham Heart Study is funded by National Institutes of Health contract N01-HC-25195. The laboratory work for this investigation was funded by the Division of Intramural Research, National Heart, Lung, and Blood Institute, National Institutes of Health, and by a Director's Challenge Award, National Institutes of Health (DL, PI). The analytical component of this project was funded by the Division of Intramural Research, National Heart, Lung, and Blood Institute, and the Center for Information Technology, National Institutes of Health, Bethesda, MD. This study utilized the computational resources of the Biowulf system at the National Institutes of Health, Bethesda, MD (http://biowulf.nih.gov). The Lifelines-Deep work was supported by the European Research Council Advanced Grant (ERC-671274 to CW), the Dutch Digestive Diseases Foundation (MLDS WO11-30 to CW), the European Union's Seventh Framework Programme (EU FP7) TANDEM project (HEALTH-F3-2012-305279 to CW), the Netherlands Organization for Scientific Research (NWO-VENI grant 916-10135 to LF and NWO VIDI grant 917-14374 to LF) and by Top Institute Food and Nutrition Wageningen (GH001 to CW). Generation of the methylation data (as part of the Biobank-based Integrative Omics Study (BIOS)) is financially supported by the Biobanking and Biomolecular Research Infrastructure of The Netherlands (BBMRI-NL), funded by the Netherlands Organisation for Scientific Research (NWO). AZ holds a Rosalind Franklin fellowship (University of Groningen).

Figure S1. BMI distribution

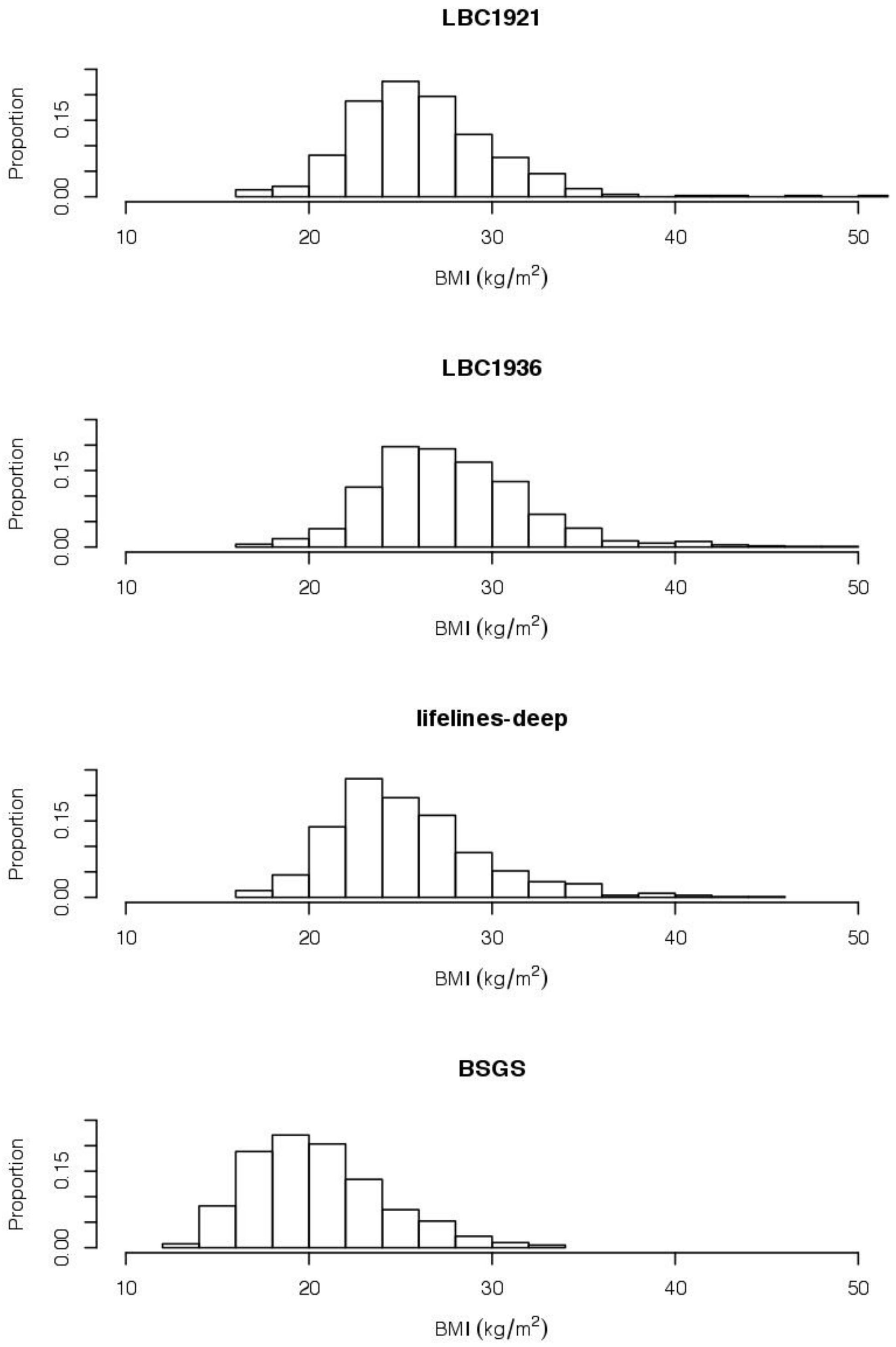

Figure S2. Height distribution

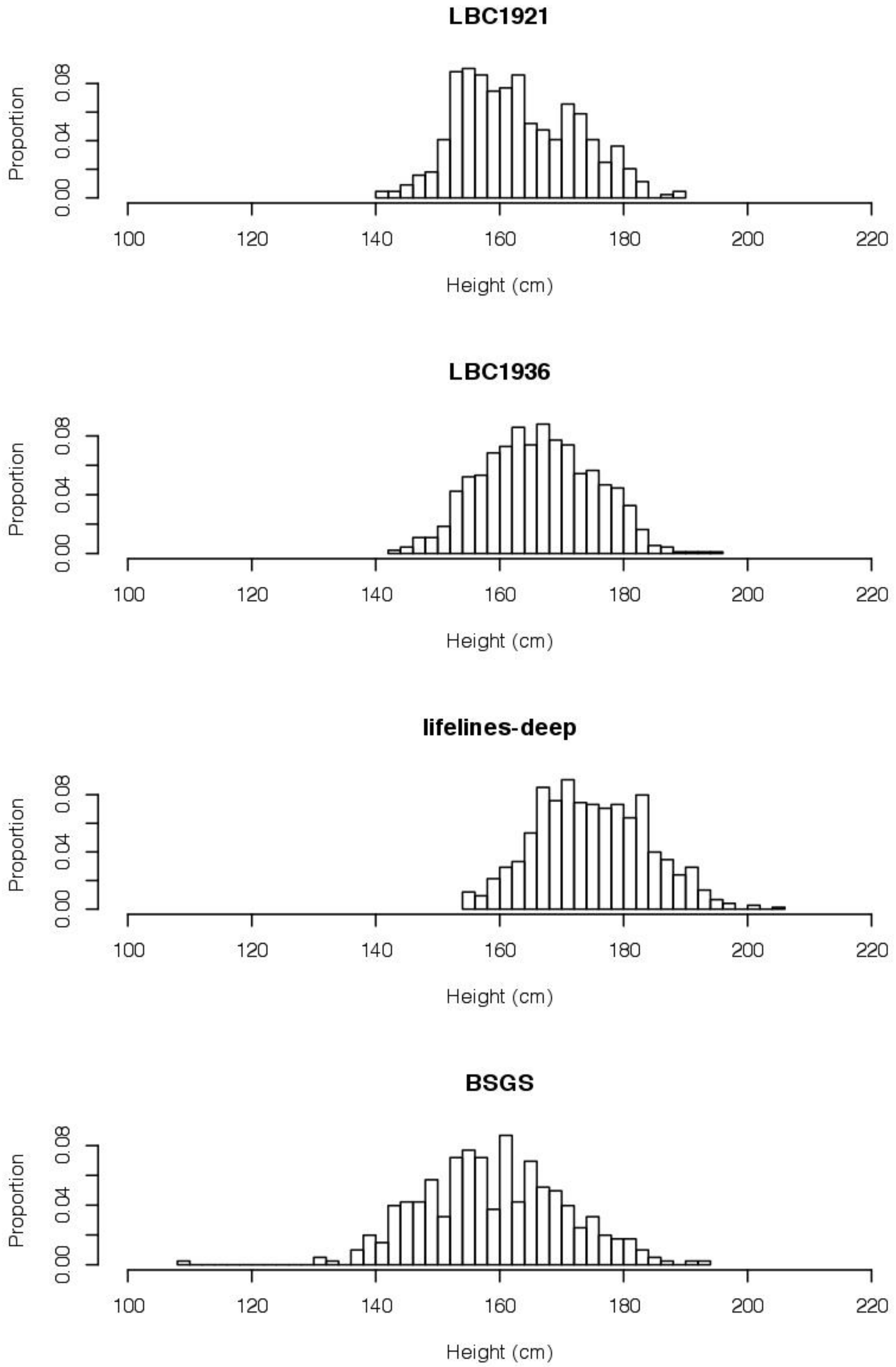

Figure S3. MWAS QQ plots. QQ plots for MWAS for BMI and height in the LBC and Lifelines cohorts.

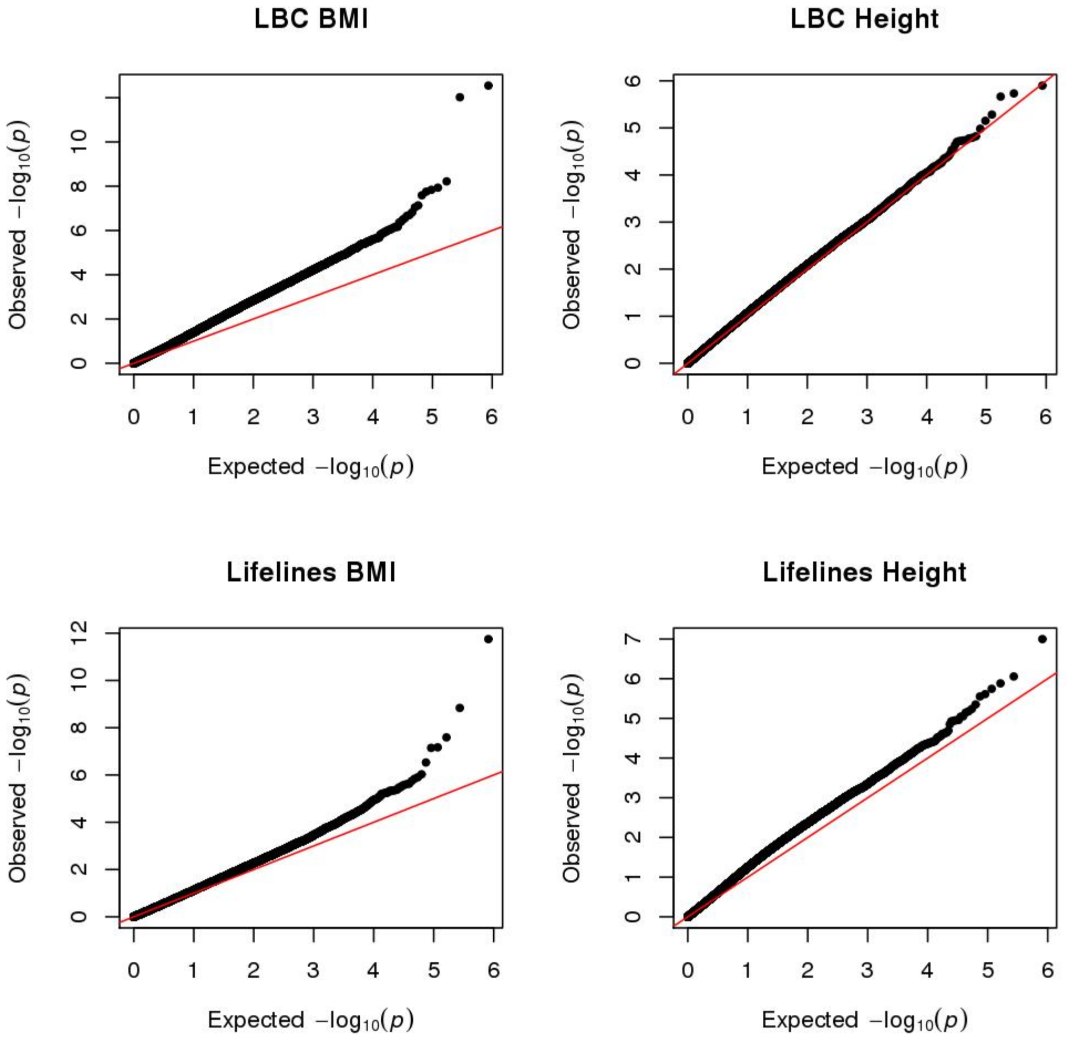

Figure S4. Framingham-based BMI methylation scores. Proportion of variance explained in sex and age-adjusted BMI where the methylation profile scores were based on CpGs identified by the Framingham MWAS. In the LBC, weights for the methylation profile scores were derived from the Lifelines-deep MWAS and *vice versa*. The plot shows the Adjusted-R^2 explained by the methylation profile score, the genetic profile score, an additive model including both scores (Methylation + Genetic), and an interaction model (Methylation * Genetic).

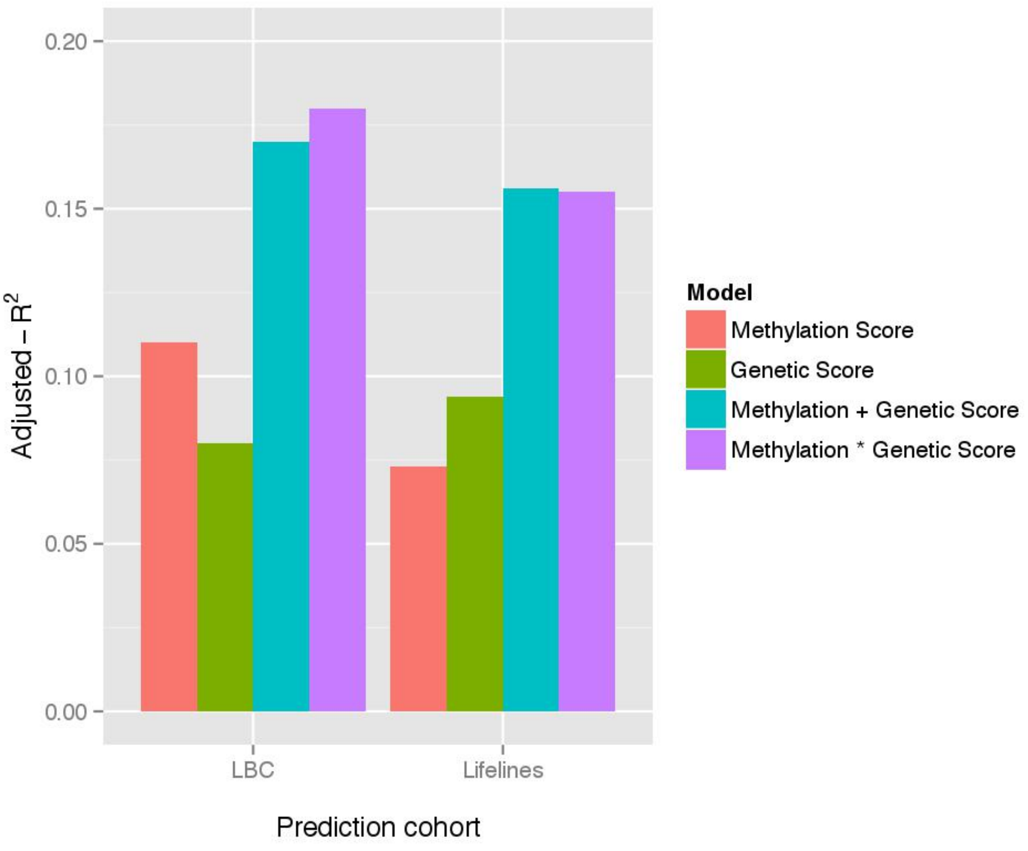

Figure S5. Proportion of variance explained in sex and age-adjusted BMI and height phenotype after correction for cell-count. The plot shows the proportion of variance (Adjusted-R^2) in sex and age-adjusted height explained by the methylation profile score, the genetic profile score, an additive model including both scores (Methylation + Genetic), and an interaction model (Methylation * Genetic).

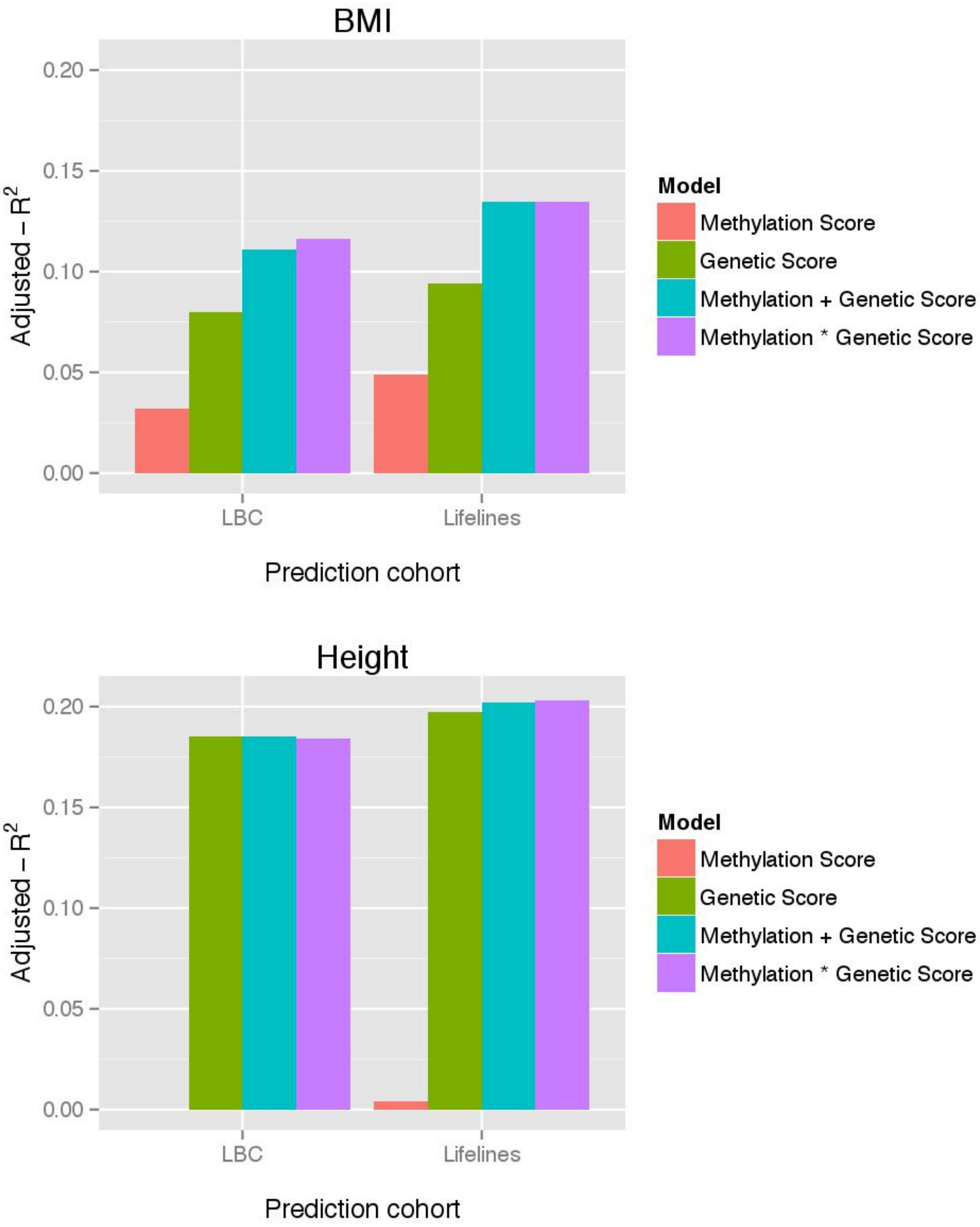

Figure S6. Causality and reverse causation.

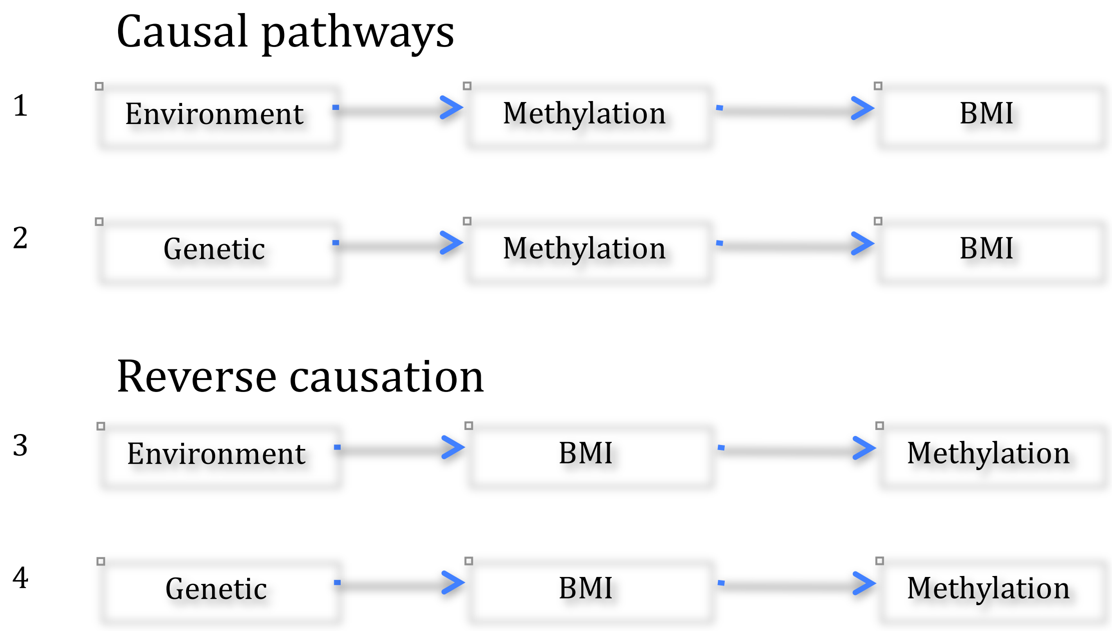

Table S1. CpG probes significantly associated with BMI in LBC and Lifelines-deep. Effect sizes are per standard deviation change in sex and age-adjusted BMI with a unit change in the transformed methylation values.

Probe	chr	BP (NCBI37)	R^2	effect	SE	P
LBC						
cg06500161	21	43656587	0.038	0.196	0.027	2.85E-13
cg11024682	17	17730094	0.036	0.192	0.027	9.56E-13
cg07094298	4	2748026	0.024	-0.157	0.027	6.04E-09
cg04011474	2	28904455	0.023	-0.154	0.027	1.16E-08
cg10192877	21	43641690	0.023	0.153	0.027	1.45E-08
cg17782974	10	104406990	0.022	0.152	0.027	1.76E-08
cg20496314	22	39759864	0.022	0.150	0.027	2.57E-08
cg07202479	1	159174162	0.020	-0.145	0.027	7.35E-08
cg09494176	4	3193868	0.020	-0.144	0.027	9.04E-08
Lifelines						
cg11024682	17	17730094	0.060	0.253	0.035	1.79E-12
cg17501210	6	166970252	0.045	-0.218	0.036	1.46E-09
cg21139312	17	55663225	0.038	0.201	0.036	2.57E-08
cg06500161	21	43656587	0.035	0.195	0.036	6.75E-08
cg26403843	5	158634085	0.036	0.195	0.036	7.14E-08

Table S2. Correlation between sex- and age-adjusted BMI and height with methylation and genetic predictors

	Methylation Predictor					
Trait	Probe selection cohort	Effect size estimation cohort	Prediction cohort	Pearson Correlation	Adjusted-R^2	Pearson correlation P-Value
BMI	Lifelines	Lifelines	LBC	0.26	0.069	$<1x10^{-15}$
BMI	LBC	LBC	Lifelines	0.22	0.049	$6.685x10^{-10}$
BMI	Framingham	Lifelines	LBC	0.33	0.11	$<1x10^{-15}$
BMI	Framingham	LBC	Lifelines	0.27	0.073	$3.952x10^{-14}$
Height	Lifelines	Lifelines	LBC	0.06	0.0031	0.02
Height	LBC	LBC	Lifelines	0.09	0.0076	0.0096

	Genetic Predictor					
Trait	Probe selection cohort	Effect size estimation cohort	Prediction cohort	Pearson Correlation	Adjusted-R^2	Pearson correlation P-Value
BMI	GIANT 2015	GIANT 2015	LBC	0.28	0.08	$<1x10^{-15}$
BMI	GIANT 2015	GIANT 2015	Lifelines	0.31	0.094	$<1x10^{-15}$
Height	GIANT 2014	GIANT 2014	LBC	0.43	0.19	$<1x10^{-15}$
Height	GIANT 2014	GIANT 2014	Lifelines	0.45	0.20	$<1x10^{-15}$

Table S3. Additive vs interaction model. Proportion of variance explained in sex- and age-adjusted BMI and height by the additive genotype/methylation model versus an interaction model.

Trait	Methylation Predictor: Probe selection cohort	Methylation Predictor: Effect size estimation cohort	Prediction cohort	Model	R^2	ANOVA p-value for model comparison
BMI	Lifelines	Lifelines	LBC	Additive	0.14	**5.0×10^{-6}**
				Interaction	0.15	
BMI	LBC	LBC	Lifelines	Additive	0.13	0.35
				Interaction	0.13	
BMI	Framingham	Lifelines	LBC	Additive	0.17	**1.4×10^{-4}**
				Interaction	0.18	
BMI	Framingham	LBC	Lifelines	Additive	0.16	0.59
				Interaction	0.16	
Height	Lifelines	Lifelines	LBC	Additive	0.19	0.64
				Interaction	0.19	
Height	LBC	LBC	Lifelines	Additive	0.20	0.50
				Interaction	0.20	

Table S4. Relative contribution of methylation and genetic scores to variance in BMI. The table shows the relative contribution of the methylation and genetic profile scores to the variation in the sex and age-adjusted BMI phenotype (the ratio of the R^2 values from the methylation and genetic profile score) in the adult cohorts compared to BSGS adolescents individuals.

Methylation score derivation	Prediction cohort	Ratio of Methylation score R^2/genetic score R^2
Discovery cohort=LBC; weights=LBC	Lifelines	0.517
Discovery cohort=LBC; weights=LBC	BSGS	0.015
Discovery cohort=Framingham; weights=LBC	Lifelines	0.777
Discovery cohort=Framingham; weights=LBC	BSGS	0.583
Discovery cohort=Framingham; weights=Lifelines	LBC	1.375
Discovery cohort=Framingham; weights=Lifelines	BSGS	1.056
Discovery cohort=Lifelines; weights=Lifelines	LBC	0.863
Discovery cohort=Lifelines; weights=Lifelines	BSGS	0.694

Table S5. Effect of p-value threshold on prediction ability of methylation score

Trait	Prediction Cohort	P-value threshold	Adj R2	P-value
	LBC	0.1	0.001	0.09
	LBC	0.001	-0.0005	0.5
	LBC	1.00E-05	0.037	6×10^{-13}
	LBC	1.00E-07	0.069	$<1 \times 10^{-15}$
BMI				
	Lifelines	0.1	-0.00005	0.3
	Lifelines	0.001	-0.0006	0.5
	Lifelines	1.00E-05	-0.0007	0.5
	Lifelines	1.00E-07	0.049	7×10^{-10}
	LBC	0.1	0.002	0.0407
	LBC	0.001	0.003	0.02161
	LBC	1.00E-05	0.0003	0.225
	LBC	1.00E-07	NA	NA
Height				
	Lifelines	0.1	0.0058	0.02
	Lifelines	0.001	0.0076	0.01
	Lifelines	1.00E-05	-0.0001	0.3
	Lifelines	1.00E-07	NA	NA
